# Oscillatory Dynamics Serving Verbal Working Memory Differ in People with HIV and Are Linked To Disease Duration

**DOI:** 10.1007/s11481-025-10235-0

**Published:** 2025-08-19

**Authors:** Kellen M. McDonald, Seth D. Springer, Mikki Schantell, Ryan Glesinger, Lucy K. Horne, Hannah J. Okelberry, Jason A. John, Anna T. Coutant, Madelyn P. Willett, Hallie J. Johnson, Rachel K. Spooner, Tony W. Wilson

**Affiliations:** 1 Institute for Human Neuroscience, Boys Town National Research Hospital, Boys Town, NE USA; 2 Department of Pharmacology and Neuroscience, Creighton University, Omaha, NE USA; 3 College of Medicine, University of Nebraska Medical Center (UNMC), Omaha, NE USA

**Keywords:** PWH, Magnetoencephalography, MEG, Alpha, Beta, Anterior cingulate cortex, Superior temporal gyrus

## Abstract

**Background:**

Medical advances have greatly improved the quality of life and extended the longevity of people with HIV (PWH). However, many PWH still develop neurocognitive deficits even in the presence of effective viral suppression, with impairments in verbal working memory (VWM) being among the most common. While previous neuroimaging studies have demonstrated altered neural responses during VWM, the underlying temporal dynamics and their relation to clinical indices of HIV remain poorly understood.

**Hypothesis:**

PWH will have altered neural oscillatory dynamics in brain regions supporting VWM compared to controls, above and beyond the effect of age, and these oscillatory differences will scale with clinical indices of HIV.

**Methods:**

166 participants (77 virally-suppressed PWH, 89 demographically-matched controls) completed a VWM task during magnetoencephalography, which was separated into encoding and maintenance phases. Whole-brain, mixed-model ANCOVAs were performed to assess the effects of HIV status on neural dynamics controlling for age.

**Results:**

PWH performed significantly worse on the task compared to controls. During encoding, there was a significant interaction of group-by-time window, such that PWH had significantly weaker alpha/beta oscillations in the left inferior frontal, superior temporal, and anterior cingulate relative to controls. Further, weaker activity in the anterior cingulate scaled with increased disease duration. PWH also displayed weaker alpha/beta oscillations during maintenance in frontal, temporal, parietal, anterior cingulate, and cerebellar cortices.

**Conclusions:**

PWH exhibited weaker task-related oscillatory activity during VWM, which was associated with disease duration in the anterior cingulate. Overall, these findings suggest that HIV modulates the neural dynamics underlying VWM, with progressive effects in some areas.

**Graphical abstract:**

**Figure:**
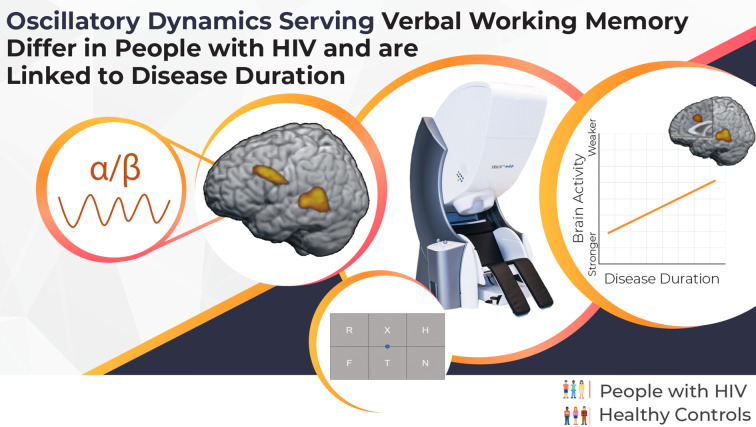
People with HIV and matched healthy controls completed a verbal working memory paradigm during magnetoencephalography. In addition to people with HIV exhibiting weaker task-related oscillatory activity compared to controls, alpha/beta activity in the anterior cingulate significantly scaled with time since HIV diagnosis

**Supplementary Information:**

The online version contains supplementary material available at 10.1007/s11481-025-10235-0.

## Introduction

The advent of combination antiretroviral therapies (cART) greatly improved the longevity and health outcomes of people with HIV (PWH; Gakhar et al. 2013; Xia et al. 2022), but cognitive decline continues to disproportionately affect this population and have a negative impact on their daily life (Davies et al. 2019; Heaton et al. 2004). Thus, there is an increasing need to understand the underlying mechanisms and neuronal origins of these cognitive impairments.

Working memory (WM) is one of the cognitive domains most commonly affected in PWH (Rowe et al. 2021; Stout et al. 1995; Wilson et al. 2017). It is defined in Baddeley and Hitch’s model as a system for the temporary storage of information that is fundamental to cognitive processing, consisting of a central executive component that oversees several subcomponents specific to the type of information being stored (Baddeley 2003; Baddeley and Hitch 1974). In the context of verbal WM (VWM), one such subcomponent is the left-lateralized phonological loop, consisting of the inferior frontal and superior temporal cortices. This system enables verbal information to be temporarily stored and supports the rehearsal of phonological information to help keep memory representations active and thereby aid in retention (Logie et al. 2003). As such, numerous studies have identified the phonological loop as an essential component of VWM (Cohen et al. 2018; Logie et al. 2003; Proskovec et al. 2016, 2019; Springer et al. 2023; Trost and Gruber 2012; Vallar et al. 1997). Importantly, deficits in the phonological loop component have been linked to significant impairments in language processing and comprehension (Baddeley 2003; Lauro et al. 2010). Mechanistic expansions of Baddeley and Hitch’s model have suggested that WM abilities rely on persistent cell activation in task-related regions, with such persistent activity being critical to the maintenance of active memory representations after the stimulus has been removed (Curtis and D’Esposito 2003; Funahashi et al. 1989; Goldman-Rakic 1995).

VWM can be separated into three phases. The first, *encoding*, involves decoding the sensory input (i.e., visual or auditory signals) and loading the information into a temporary storage. The subsequent *maintenance* phase encompasses the rehearsal of the encoded information to help maintain representations in short-term storage, while this information is recalled and utilized to achieve a cognitive goal in the *retrieval* phase (Heinrichs-Graham and Wilson 2015). To examine each of these three phases, previous magnetoencephalography (MEG) studies have used a modified Sternberg WM paradigm to directly quantify population-level neural oscillatory activity during these distinct phases of VWM ability (McDermott et al. 2016; Wiesman et al. 2016; Wilson et al. 2017; Proskovec et al. 2016, 2019; Killanin et al. 2022; Springer et al. 2023). In these studies, a sharp increase in theta power, often observed between 3 and 7 Hz, is generally seen at the start of encoding in the occipital cortices, in line with the notion that such theta responses reflect early visual processing (Killanin et al. 2024). This is quickly followed by a strong, sustained decrease relative to baseline in alpha/beta power, usually extending from about 12 Hz up to 20 Hz. Such alpha/beta activity then increases in power or rebounds during the maintenance phase, especially in posterior regions of the parietal and occipital cortices (Wiesman et al. 2016; Wilson et al. 2017; Proskovec et al. 2016, 2019; Killanin et al. 2022; Springer et al. 2023). In support of these previous findings, intracranial investigations of WM have shown significant oscillations in the theta, alpha/beta, and gamma frequency ranges (Bastos et al. 2015; Salazar et al. 2012; Zhang et al. 2018), with some suggesting that such alpha and/or beta oscillations reflect top-down processing and inhibitory control in support of executive functioning (Miller et al. 2018).

Executive functions serving memory and attention have been found to be particularly vulnerable to the effects of HIV, as well as healthy aging (Cohen et al. 2018; Davies et al. 2019; Lew et al. 2018, 2023; Wilson et al. 2017). Studies utilizing MEG have further shown that these deficits in executive functions are associated with aberrant neuronal activity, particularly in the higher-order prefrontal regions but also primary sensory cortices of the brain (Lew et al. 2018; Meehan et al. 2023; Wilson et al. 2017, 2019). One MEG study evaluating VWM and aging reported age-related alterations in fronto-temporal and parieto-occipital alpha/beta activity during the encoding and maintenance phases (Proskovec et al. 2016). A later MEG study using this same VWM paradigm found that PWH performed significantly worse (i.e., less accurate) and exhibited accentuated oscillatory alpha oscillations in left-lateralized areas commonly associated with VWM processing, including the left supramarginal and inferior frontal gyri, as well as attenuated alpha oscillations in right hemispheric homologue regions during memory maintenance compared to healthy aging controls (Wilson et al. 2017). The authors suggested that their data supported the compensation-related utilization of neural circuits hypothesis (CRUNCH; Reuter-Lorenz and Cappell 2008), in that the healthy aging controls leveraged homologue cortices in the right hemisphere to maintain high performance, whereas the PWH did not and instead relied on hyper-activity in left hemispheric regions, which ultimately led to degraded task performance. Overall, such a pattern may indicate accelerated aging in PWH, meaning that the normal effects of older age on cognitive processing may impact PWH sooner (Arif et al. 2020; Gross et al. 2016; Lew et al. 2020, 2021; Schantell et al. 2022). However, caution with this interpretation is warranted, as age was not included in the statistical model, and the overall sample size was limited (i.e., *N* < 40 total; Wilson et al. 2017). Thus, while it is evident that aging and HIV significantly affect the neural dynamics serving VWM, much remains to be discovered.

Impairments in VWM greatly impact one’s ability to successfully perform daily tasks such as remembering needed items while shopping, adhering to medication regimens, and managing finances (Heaton et al. 2004). VWM deficits are also associated with reduced comprehension, which can impair effective communication (Payne and Stine-Morrow 2017). However, despite such importance to independent living, it remains unclear how the neural dynamics serving VWM are affected by HIV status above and beyond the effects of normal aging, as well as the extent to which clinical indices of HIV disease are coupled to such altered oscillatory dynamics. To address these gaps, we enrolled a large sample of virally-suppressed PWH and demographically-matched controls who completed a validated verbal Sternberg WM task during MEG. We hypothesized that PWH would perform worse on the VWM paradigm and exhibit aberrant theta and alpha/beta oscillatory activity, above and beyond the effects of healthy aging, in key brain regions supporting VWM processing during both the encoding and maintenance phases. We also hypothesized that this activity would be significantly related to task performance and clinical indices of HIV disease.

## Methods

### Participants

We enrolled 166 participants, including 77 PWH and 89 controls between the ages of 20 and 66 years (mean age: 45.7 years). The two groups included in the full analysis did not statistically differ in age, sex, race, ethnicity, tobacco use, or alcohol use (Table 1). All PWH were receiving effective antiretroviral treatment and were virally-suppressed at enrollment, defined as < 50 copies/mL. All controls were confirmed to be seronegative for HIV using the OraQuick ADVANCE^®^ Rapid HIV-1/2 Antibody Test at the time of neuropsychological testing. Exclusionary criteria included any medical illness affecting CNS function, any neurological or severe psychiatric disorder, current substance use disorder, history of head trauma, and any non-removable metal implants that would affect MEG data acquisition or be an MRI safety concern. The Institutional Review Board (IRB) approved the study protocol and written informed consent was obtained from each participant after a full description of the study.

### Neuropsychological Assessment

Cognitive function was assessed by a comprehensive neuropsychological battery that evaluated functionality across seven domains. The battery included tests for *learning* (Delis et al. 2000; Wechsler 1997b), *memory* (Wechsler 1997b), *processing speed* (Comalli et al. 1962; Heaton 2004; Wechsler 1997a), *attention* (Delis et al. 2000; Wechsler 1997a), *language* (Heaton 2004), *executive function* (Comalli et al. 1962; Heaton 2004), and *motor dexterity* (Heaton 2004; Kløve 1963). Demographically corrected scores for each assessment were obtained using published normative data (Comalli et al. 1962; Delis et al. 2000; Heaton 2004; Kløve 1963; Wechsler 1997a) and were transformed to z-scores. Domain composite scores were computed by averaging the z-scores of tests that comprised each respective cognitive domain.

### Task Paradigm

**Fig. 1:**
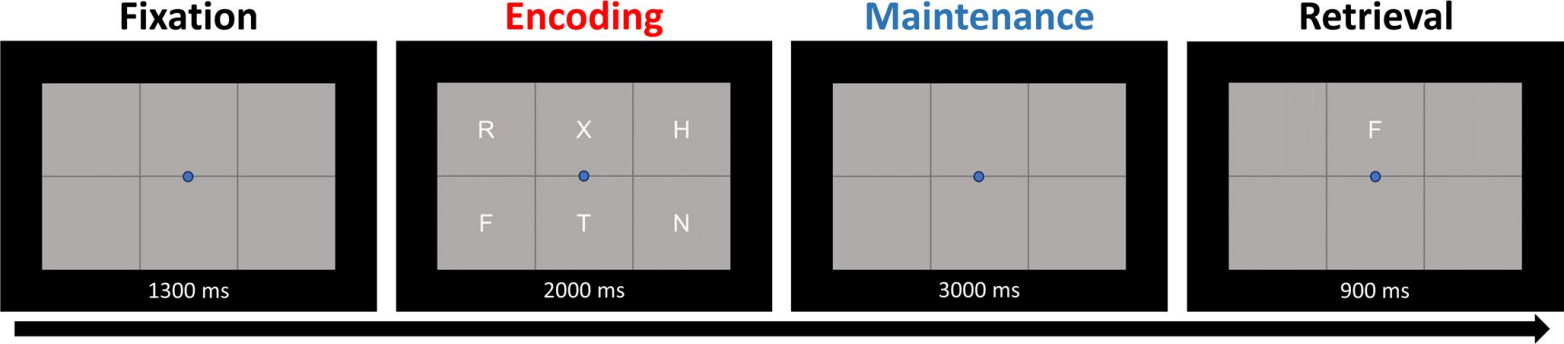
Modified Sternberg working memory paradigm. After the fixation period, the encoding period began with six letters appearing for 2.0 s. The letters then disappeared for 3.0 s (maintenance phase) and participants were required to maintain the letters in memory. A probe then appeared (retrieval phase) and participants were instructed to respond via button press as to whether the probe was present (in set) or not (out of set) during the encoding phase

Participants completed a modified Sternberg-type working memory task (Fig. 1), which has been used in many previous MEG studies (Embury et al. 2018, 2019; Heinrichs-Graham and Wilson 2015; Proskovec et al. 2019; Springer et al. 2023; Wiesman et al. 2016). A centrally presented fixation point embedded in a 3 × 2 grid was shown for 1.3 s (i.e., baseline period), followed by an array of six consonants at fixed locations within the grid for 2 s (i.e., encoding period). The letters then disappeared from the array and the empty grid remained on screen for 3 s (i.e., maintenance phase), which was followed by the appearance of a probe letter for 0.9 s (i.e., retrieval phase). Participants were instructed to respond via button press with their right index finger if the probe letter was part of the previous array or with their middle finger if it was not. Participants completed a total of 128 trials, equally split and pseudorandomized between in- and out-of-set trials, with a run time total of approximately 15 min.

### MEG Data Acquisition

Our MEG data acquisition, structural coregistration, preprocessing, and sensor- and source-level analyses closely followed previous analysis pipelines (see Wiesman and Wilson 2020). Recordings were conducted in a one-layer magnetically shielded room with active shielding engaged for environmental noise compensation. Neural data were recorded at 1 kHz with an acquisition bandwidth of 0.1–330 Hz using a 306-sensor Elekta/MEGIN MEG system (Helsinki, Finland) equipped with 204 planar gradiometers and 102 magnetometers. Analysis focused on the planar gradiometers. Participants were continuously monitored during data acquisition via an audio-video feed from inside the MEG room. Each dataset was corrected for head motion and subjected to noise reduction using the signal space separation method with a temporal extension and the following parameters (correlation limit: 0.950; correlation window duration: 6 s; Taulu and Simola 2006). For motion correction, the position of the head throughout the recording was aligned to the individual’s head position when the recording was initiated.

### Structural MRI and MEG Coregistration

Prior to MEG acquisition, four coils were attached to each participant’s head and localized along with fiducial and scalp surface points using a three-dimensional digitizer (Fastrak, Polhemus Navigator Sciences, Colchester, VT, USA). Once the participant was positioned for MEG recording, an electric current with a unique frequency label (e.g., 322 Hz) was fed into each coil, inducing a measurable magnetic field and therefore allowing each coil to be localized in reference to the sensors throughout the MEG recording session. Since coil locations were also known in head coordinates, all MEG measurements could be transformed into a common coordinate system. With this coordinate system, each participant’s MEG data were co-registered with structural T1-weighted MRI data using BESA MRI (Version 2.0) prior to source-space analysis. Structural MRI data were acquired using a Siemens Prisma 3T scanner (Siemens Medical Solutions) with a 64-channel head coil and a MP-RAGE sequence with the following parameters: TR: 2300 ms; TE = 2.98 ms; flip angle = 9°; FOV = 256 mm; slice thickness = 1.00 mm; voxel size = 1 × 1 × 1 mm. The resulting images were aligned parallel to the anterior/posterior commissures and transformed into standardized space. Following beamformer analyses, each participant’s 4.0 × 4.0 × 4.0 mm functional images were also transformed into standardized space using the transform that was previously applied to the structural MRI volume and spatially resampled.

### MEG Preprocessing, Time-Frequency Transformation, and Sensor-Level Statistics

Cardiac and blink artifacts were removed from the MEG data using signal space projection (SSP), which was subsequently accounted for during source reconstruction (Uusitalo and Ilmoniemi 1997). The continuous magnetic time series was then filtered between 0.5 and 200 Hz, plus a 60 Hz notch filter, and divided into 7200 ms epochs, with the baseline extending from − 400 to 0 ms prior to the onset of the encoding phase. Epochs containing artifacts were rejected using a fixed threshold method, supplemented with visual inspection. Briefly, in MEG, the raw signal amplitude is strongly affected by the distance between the brain and the MEG sensor array, as the magnetic field strength falls off sharply as the distance from the current source increases. To account for this source of variance across participants, as well as actual variance in neural response amplitude, we used an individually determined threshold based on the signal distribution for both signal amplitude and gradient to reject artifacts. Across all participants, the average amplitude threshold was 1316.5 (SD = 421.65) fT/cm and the average gradient threshold was 559.4 (SD = 289.23) fT/(cm*ms), neither of which were significantly different by group (*p* > .05). An average of 89.91 (SD = 12.71) trials per participant (out of 128 possible trials) were used for further analysis, and this did not statistically differ by group (*p* > .05).

Following artifact rejection, the remaining epochs were transformed into the time-frequency domain using complex demodulation (Kovach and Gander 2016; Papp and Ktonas 1977). This process begins with transforming the signal into the frequency space using a discrete Fast Fourier Transform (FFT), which results in a frequency spectrum containing the same power and cross spectrum information as the original signal. Next, in a process termed heterodyning, the frequency spectrum is (de)modulated in a stepwise manner to adopt the center frequency of a series of complex sinusoids with increasing frequencies. The resulting signals are low pass filtered to reduce spectral leakage, which determines the time and frequency resolution of the resulting data. In this study, time-frequency analysis used a frequency-step of 1 Hz and time-step of 50 ms between 2 and 50 Hz. Following time-frequency transformation, spectral power estimates per sensor were averaged across trials to generate plots of mean spectral density per sensor, which were then normalized to the baseline power within each frequency bin, calculated as the mean power per 1 Hz bin during the − 400 to 0 ms time period.

To identify the oscillatory responses for imaging, a two-stage statistical approach was used to minimize Type 1 error while maintaining sensitivity. This involved a mass univariate approach based on the general linear model to assess each pixel in the sensor-level spectrograms across the entire array of gradiometers, followed by cluster-based nonparametric permutation testing to control for multiple comparisons (Ernst 2004; Maris and Oostenveld 2007). First, paired-sample t-tests against baseline were performed on each pixel per spectrogram across all participants and the output spectrograms of t-values were thresholded at *p* < .05 to define time-frequency pixels containing potentially significant deviations from baseline. The surviving pixels (*p* < .05) were then clustered with temporally and/or spectrally neighboring pixels that also survived, and cluster values were derived by summing all *t*-values of all data points within each cluster. In the second step, nonparametric permutation testing was used to derive a distribution of cluster values, and the significance level of the observed clusters from step one were tested directly using this permuted distribution, which was the result of 10,000 permutations. Based on these analyses, the clusters (i.e., time-frequency windows) that contained significant oscillatory deviations from the baseline period (at *p* < .001, corrected) across all participants were subjected to source imaging (i.e., beamforming).

### MEG Source Analysis

Oscillatory responses were imaged through a time-frequency-resolved extension of the linearly constrained minimum variance (LCMV) beamformer (Gross et al. 2001; Van Veen et al. 1997). The images were derived from the cross-spectral densities of all combinations of MEG gradiometers averaged over the time-frequency range of interest, and the solution of the forward problem for each location on a grid specified by input voxel space. This use of the cross-spectral densities is often referred to as the dynamic imaging of coherent sources approach (DICS; Gross et al. 2001). Following convention, the source power in these images was normalized per participant using a pre-stimulus baseline period of equal duration and bandwidth (Hillebrand et al. 2005). Using baseline and active periods with equal durations and bandwidths is essential as it ensures the dual state beamformer is not biased by differing amounts of data being included in the computation of one or the other period. Such images are typically referred to as pseudo-t maps, with the pseudo-t units reflecting the noise-normalized power differences (i.e., active vs. passive) per voxel. This approach generated three-dimensional, voxel-wise, participant-level maps for each time-frequency window identified in the sensor-level time-frequency analysis. To assess data quality and general response patterns, these pseudo-t maps were averaged across participants per group, generating group-wise average whole-brain maps for the selected time-frequency windows. MEG pre-processing and imaging used the Brain Electrical Source Analysis (BESA version 7.0) software.

### Statistical Analyses

Statistical analyses were performed using custom R and MATLAB (version 9.5.0) scripts, as well as SPM12 (Wellcome Centre for Human Neuroimaging; London, UK). The encoding phase of the VWM task was divided into four distinct time bins of 400 ms each, corresponding to the duration of the baseline. A whole-brain, 4 × 2 mixed model ANCOVA controlling for chronological age was used to investigate main effects and interactions across the encoding period using MATLAB (MathWorks; Natick, Massachusetts). For individual time periods (e.g., during maintenance), whole-brain, one-way ANCOVAs controlling for age were computed. Where needed, peak values from significant clusters were extracted for post-hoc testing using IBM SPSS Statistics v.29. A voxel-wise significance threshold of *p* < .005 was first used for the identification of potentially significant voxels in the whole-brain statistical maps. A cluster-extent (*k*) threshold of at least 12 contiguous voxels (i.e., >768 mm^3^ of brain tissue) was then applied to the thresholded statistical map to account for multiple comparisons, with analyses limited to the cerebellar and cortical gray matter. In other words, significant clusters were comprised of at least 12 voxels that shared sides or edges, with each voxel exceeding the *p* < .005 threshold. The peak voxel of each significant cluster was then extracted and used in Pearson correlational analyses with behavioral task performance (i.e., reaction time and accuracy) and clinical HIV metrics (i.e., time since diagnosis, length of time on ART, and CD4 count) using SPSS. Task performance and neuropsychological assessment group comparisons were performed via independent samples t-test using SPSS.

## Results

### Participant Characteristics

Of the 166 enrolled participants, three controls were excluded for issues with task administration (i.e., technical errors), eight participants were excluded for poor task performance (i.e., low accuracy) and/or low trial count after artifact rejection (two controls and six PWH), four participants were excluded for excessive movement during MEG (two controls and two PWH), and one control participant withdrew from the study. Thus, the final sample consisted of 81 healthy controls and 69 PWH, with ages ranging from 20 to 66 years old. The two groups were closely matched on important demographic variables such as age, sex, race, and ethnicity. Table 1 provides a detailed breakdown of participant demographics.

**Table 1:** Participant demographics

	Controls *n* = 81	PWH *n* = 69	*p*-value
Age (years)	45.4 (13.1)	46.7 (12.3)	0.55
Sex (% female)	21.0%	23.2%	0.75
AUD	8.6%	7.2%	0.75
Tobacco Use	3.7%	2.9%	0.58
Race			0.71
White	79.0%	81.2%	
Black or African American	11.1%	10.1%	
Pacific Islander	0%	0%	
Asian	6.2%	2.9%	
American Indian/Alaska Native	0%	1.4%	
More than 1 race	3.7%	4.3%	
Ethnicity			0.77
Hispanic	7.4%	8.7%	
Not Hispanic	92.6%	91.3%	
Neuropsychological Assessments (z-scores)			
Learning	0.66	0.39	0.06
**Memory**	0.70	0.42	**0.03**
Processing Speed	0.06	-0.08	0.46
**Attention**	0.34	-0.02	**0.002**
Executive Function	0.26	0.13	0.55
Language	0.02	0.07	0.69
**Motor**	0.07	-0.52	**0.015**
HIV Clinical Metrics			-
Time Since Diagnosis (years)	-	13.7 (8.7)	
Length of Time on ART (years)	-	11.6 (7.5)	
Current CD4 Count	-	774.9 (359.5)	

Note. Percentages shown for sex, alcohol use disorder (AUD), tobacco use, race, and ethnicity (*x*^2^ used for statistics). Means and SDs are provided for age and HIV clinical metrics. Average z-scores shown for neuropsychological assessment domains. Groups did not differ on moderate to severe alcohol use or mild to moderate tobacco use. Items in bold indicate that the measure was significantly different between groups

### Neuropsychological Assessment and Task Performance

**Fig. 2:**
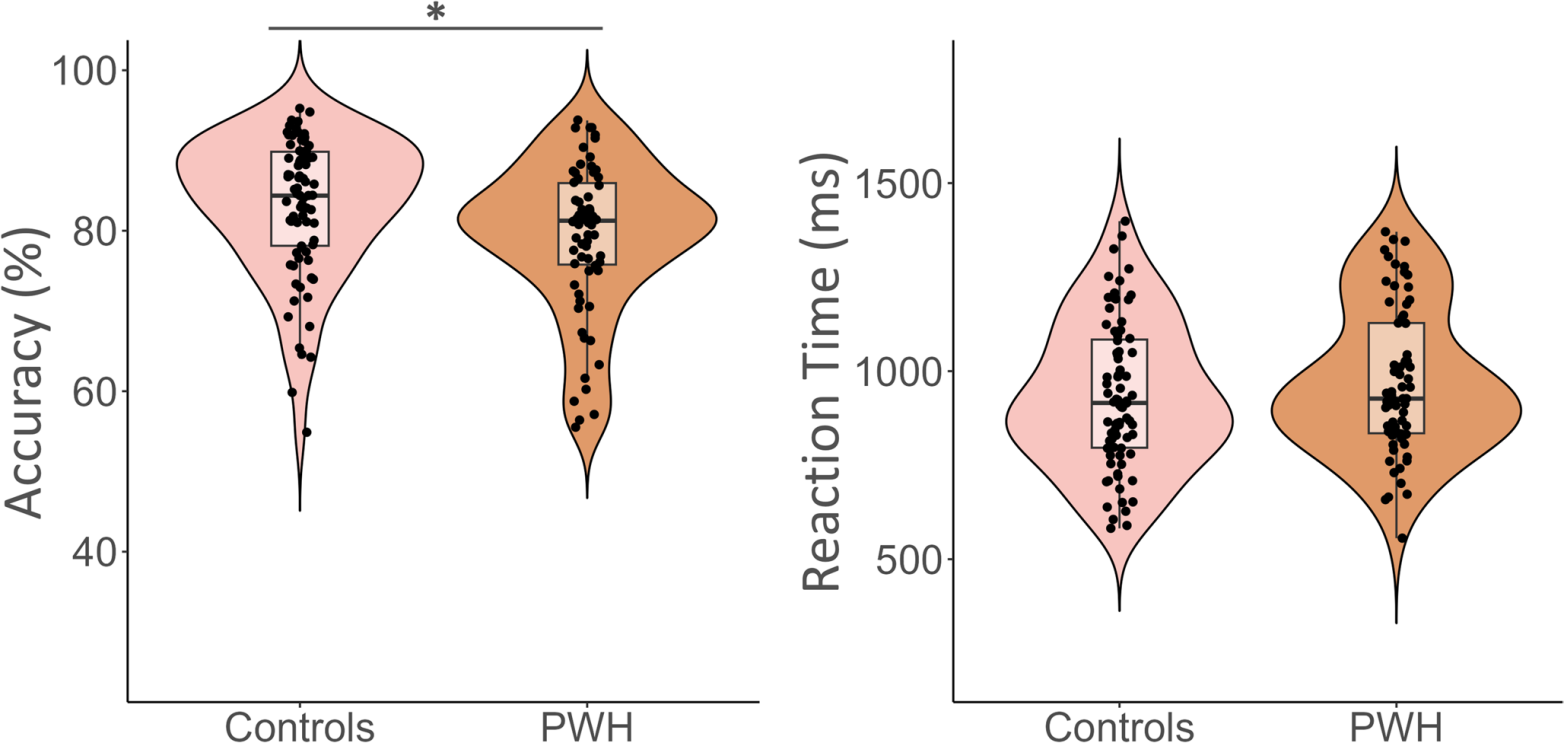
Behavior performance on the verbal working memory paradigm. Control participants were significantly more accurate than people with HIV (PWH) on the working memory task. There were no significant group differences in reaction time. *p < .05

Performance on assessments for learning, processing speed, executive function, and language did not significantly differ by group (Table 1). Attention (*t* = 3.13, *p* = .002), motor (*t* = 2.45, *p* = .015), and memory (*t* = 2.14, *p* = .034) z-scores were significantly different by group, with PWH performing significantly worse. Control participants were also significantly more accurate on the VWM paradigm compared to PWH *(t* = 2.79, *p* = .006; Fig. 2). However, there was no difference in reaction time between groups. To further validate the paradigm’s assessment of VWM, we examined relationships between individual memory test raw scores and paradigm performance. Higher raw scores on the WAIS-IV Digit Span Test (*r* = .40, *P*_*corrected*_ < .001) and WAIS-IV Letter-Number Sequencing Test (*r* = .42, *P*_*corrected*_ < .001) were associated with higher VWM accuracy after adjusting for multiple comparisons using a Bonferroni correction. Based on these findings, our MEG task reliably assessed VWM.

### Sensor-Level Analysis

**Fig. 3:**
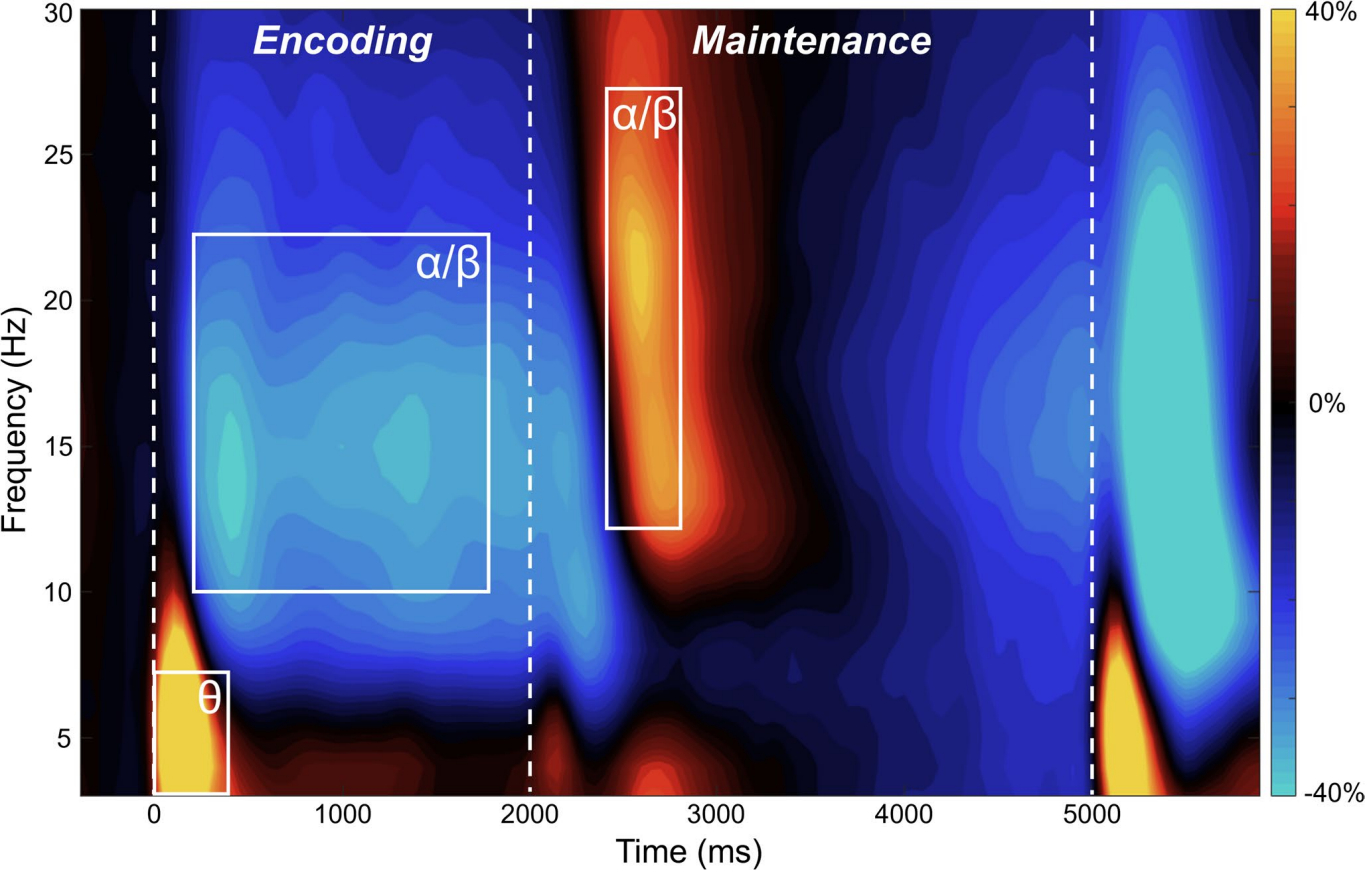
MEG sensor-level oscillatory responses during verbal working memory processing. Grand-averaged time-frequency spectrogram from a sensor near parieto-occipital areas. Frequency (Hz) is shown on the y-axis and time (ms) on the x-axis. A color scale bar is shown to the right of the spectrogram, which shows percent power change relative to the baseline period (-400 to 0 ms)

Sensor-level analyses collapsed across both groups revealed several significant time-frequency windows during the encoding and maintenance phases in all participants (Fig. 3). Specifically, a significant increase in power relative to baseline was observed in the theta range (3–7 Hz) at the start of the encoding phase (0-400 ms). We also observed a significant decrease in alpha/beta (10–22 Hz) power during encoding (200–1800 ms), as well as a significant increase in alpha/beta (12–27 Hz) power during early maintenance (2400–2800 ms). All identified windows were significant at *p* < .001, corrected. These data are broadly in line with previous reports examining working memory using MEG (Arif et al. 2024; Heinrichs-Graham and Wilson 2015; Killanin et al. 2024; Proskovec et al. 2016, 2019; Springer et al. 2023; Wiesman et al. 2016). Next, we divided the encoding window of alpha/beta oscillatory responses into non-overlapping 400 ms bins, producing four equal alpha/beta encoding windows. All significant time windows were subjected to a functional mapping analysis using a beamforming approach to examine the spatiotemporal dynamics of VWM in PWH and healthy controls.

### Functional Mapping Analysis

**Fig. 4:**
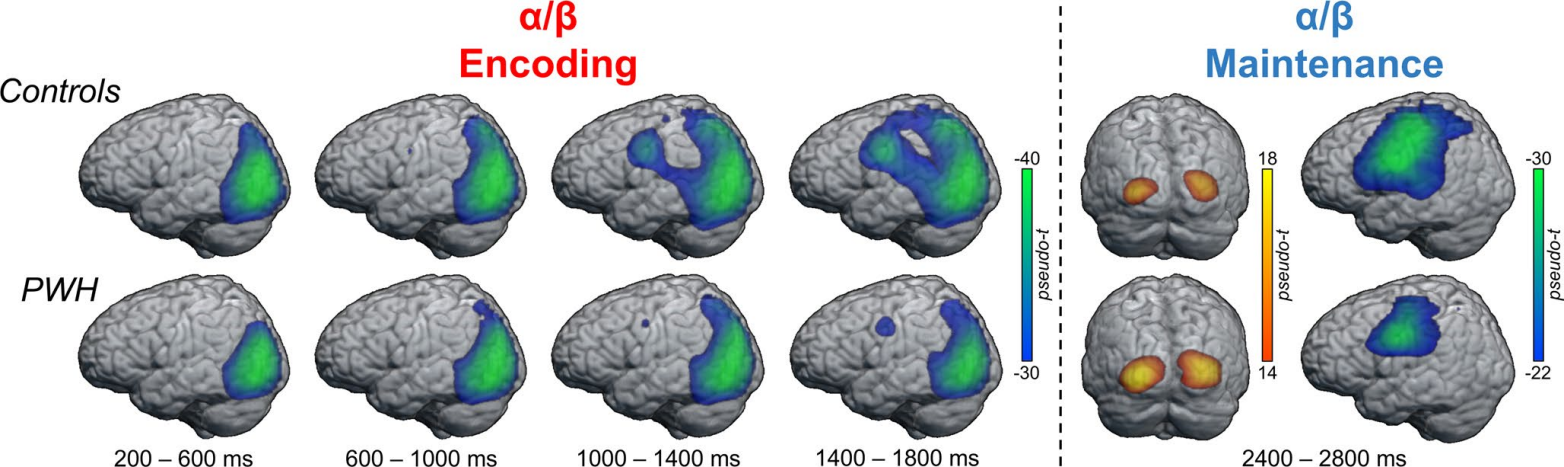
Oscillatory alpha/beta dynamics across the encoding and maintenance periods by group. The alpha/beta encoding period was divided into four equal time bins and averaged within group. Both groups exhibited a strong, sustained decrease in alpha/beta power relative to the baseline in left occipitotemporal cortices, spreading anterior to include supramarginal and fronto-parietal regions during later encoding and the maintenance period. A robust increase in alpha/beta power relative to the baseline was also observed during the maintenance period in the bilateral occipital cortices. Oscillatory maps of healthy controls are displayed along the top row, while those from people with HIV (PWH) are shown in the bottom row. Scale bars are shown to the right in each set of images

The previously mentioned sensor-level time-frequency bins of interest for theta and alpha/beta activity were imaged using a time-frequency resolved beamformer. Whole-brain maps per participant were then averaged within both groups to visualize the neural oscillations during each time bin (Fig. 4). In the alpha/beta range, there was a strong sustained decrease in power at the start of the encoding period in occipital and posterior regions in both groups. This robust oscillatory activity gradually spread anterior across the left hemisphere during the remainder of the encoding period and slightly into the maintenance phase, eventually reaching left-lateralized language regions such as the supramarginal and inferior frontal gyri. About 400 ms into the maintenance phase (2400–2800 ms), a sharp increase in alpha/beta power was observed in the bilateral visual cortices. Theta activity across the encoding period was generally limited to bilateral visual regions within occipital cortices (Supplemental Figure S1).

### Group Differences in Oscillatory Activity

**Fig. 5:**
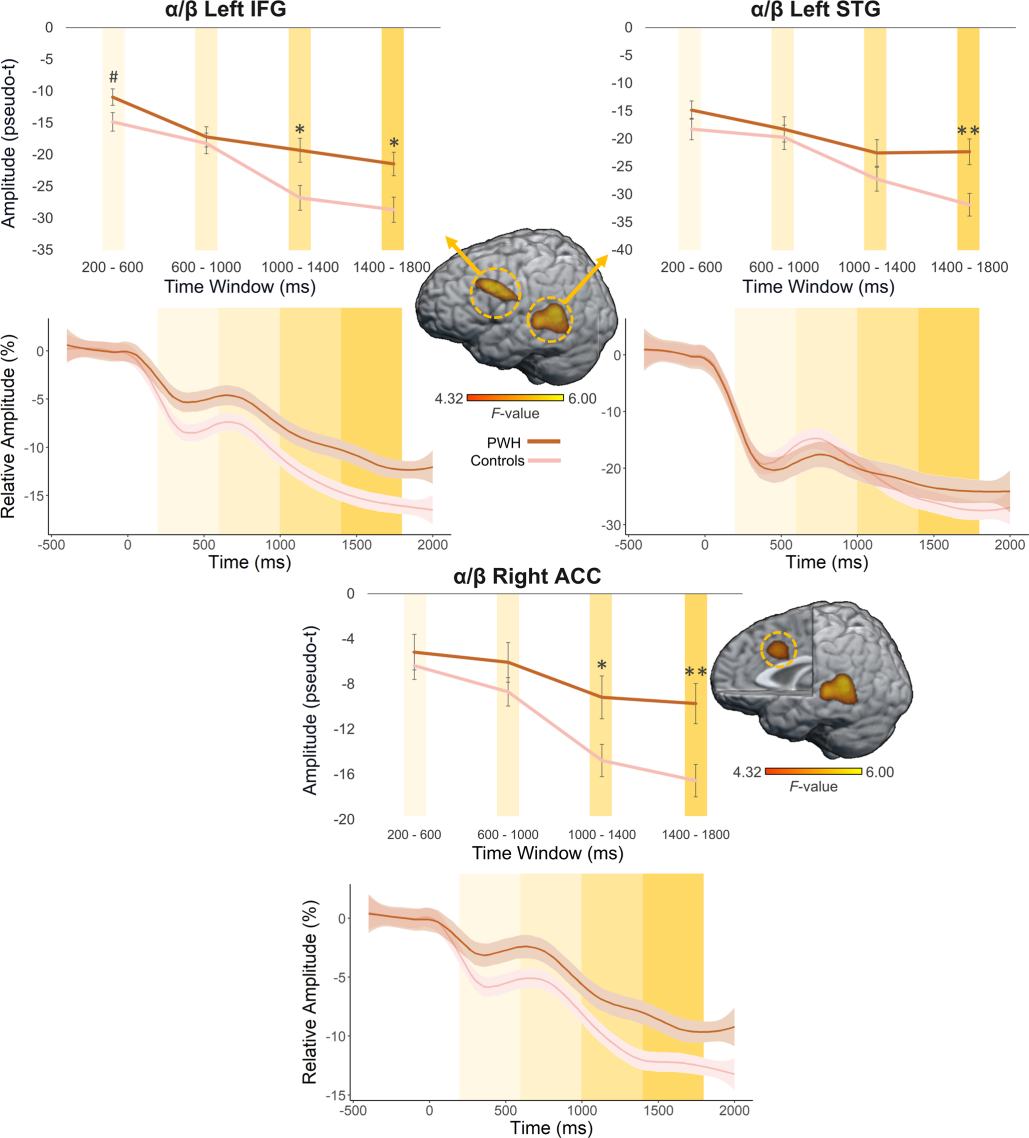
HIV status modulates the oscillatory dynamics during working memory encoding in brain regions implicated in the phonological loop and executive function. Whole-brain mixed-modal ANCOVAs controlling for age revealed significant HIV-status-by-encoding window interactions in the alpha/beta range in the left inferior frontal (top left) and superior temporal gyri (top right), as well as the right anterior cingulate cortex (ACC; bottom). This effect was driven by the last two encoding windows in the inferior frontal gyrus and ACC, and by the last window in the superior temporal gyrus. The extracted time series for each region is shown to visualize the dynamic changes in relative amplitude across encoding in each group, with shading representing the standard error within each group. Note that stronger alpha/beta oscillations are more negative relative to the baseline. Color bars of the corresponding F-values are depicted below the brain maps. #*p* = .053, *p < .05, **p < .005

**Fig. 6:**
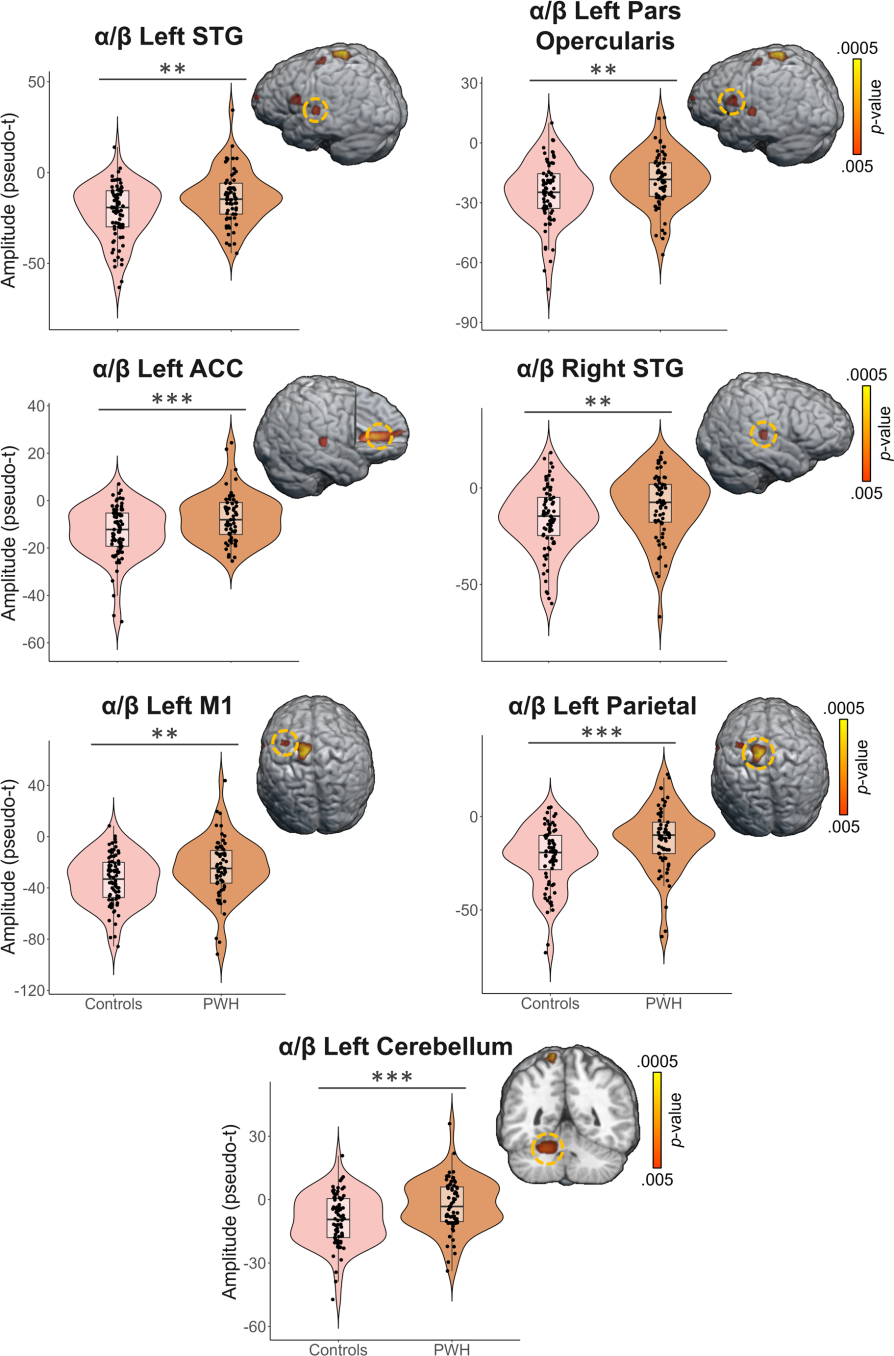
HIV status modulates the alpha/beta oscillations during memory maintenance. Whole-brain, one-way ANCOVA controlling for age revealed significant differences by group during the maintenance period in the alpha/beta range in the (top row) left superior temporal gyrus (STG) and left pars opercularis of the inferior frontal gyrus, left anterior cingulate cortex (ACC) and right STG (second row), as well as the (third row) left primary motor cortex (M1) and left parietal, and finally the left cerebellum (fourth row). Each statistical brain map is accompanied by a violin plot depicting group differences in response strength. Color bars of corresponding p-values are depicted to the far right of each row. **p < .005, ***p < .001

To statistically examine the effects of HIV status on the neural dynamics underlying VWM, we conducted whole-brain, mixed-modal ANCOVAs controlling for age. During the encoding period, we found significant group-by-time window interactions, as well as main effects of group and window. The interaction maps indicated that alpha/beta responses were significantly weaker (i.e., less negative relative to baseline) in the left inferior frontal gyrus (IFG; *F* = 6.61, *p* < .005, corrected), left superior temporal gyrus (STG; *F* = 6.84, *p* < .005, corrected), and right anterior cingulate cortex (ACC; *F* = 5.52, *p* < .005, corrected) in PWH compared to controls during specific time periods of the encoding window (Fig. 5). Post-hoc analyses using independent samples t-tests revealed significant group differences in the third encoding window in the IFG (*t* = -2.72, *p* = .007) and ACC (*t* = -2.37, *p* = .019), as well as the final encoding window within the IFG (*t* = -2.61, *p* = .010), ACC (*t* = -3.00, *p* = .003), and STG (*t* = -3.09, *p* = .002). The difference in response strength also approached significance in the first encoding window in the left IFG (*t* = -1.95, *p* = .053). To better visualize the dynamics, we extracted peak voxel time series to show neural activity across the encoding period, reported as percent changes relative to baseline (Fig. 5). In addition, main effects of group were observed in the left parietal (*F* = 11.61, *p* < .005, corrected) and primary motor cortex (*F* = 10.50, *p* < .005, corrected). Main effects of time window were also observed across extended regions of the cortex, but these were not further examined as they generally reflected the posterior to anterior wave of spreading activation found in many higher-order visual tasks. For the theta encoding response, a one-way ANCOVA was utilized to determine the effect of group on theta oscillations controlling for age. Results for this analysis, depicting stronger theta in the occipital cortices in controls compared to PWH (*F* = 10.19, *p* < .005, corrected), can be found in Supplemental Figure S2. A similar approach was taken to assess the single time window alpha/beta maintenance response, which revealed significant group differences in the left STG (*F* = 10.30, *p* < .005, corrected), left pars opercularis area of the IFG (*F* = 9.07, *p* < .005, corrected), ACC (*F* = 12.29, *p* < .005, corrected), cerebellum (*F* = 11.50, *p* < .005, corrected), left primary motor cortex (M1; *F* = 11.20, *p* < .005, corrected), left parietal (*F* = 17.95, *p* < .005, corrected), and the right STG (*F* = 9.15, *p* < .005, corrected), with PWH displaying significantly weaker (i.e., less negative relative to baseline) neural oscillations compared to control participants (Fig. 6).

### Neurobehavioral Relationships & Links To Indices of HIV Disease

**Fig. 7:**
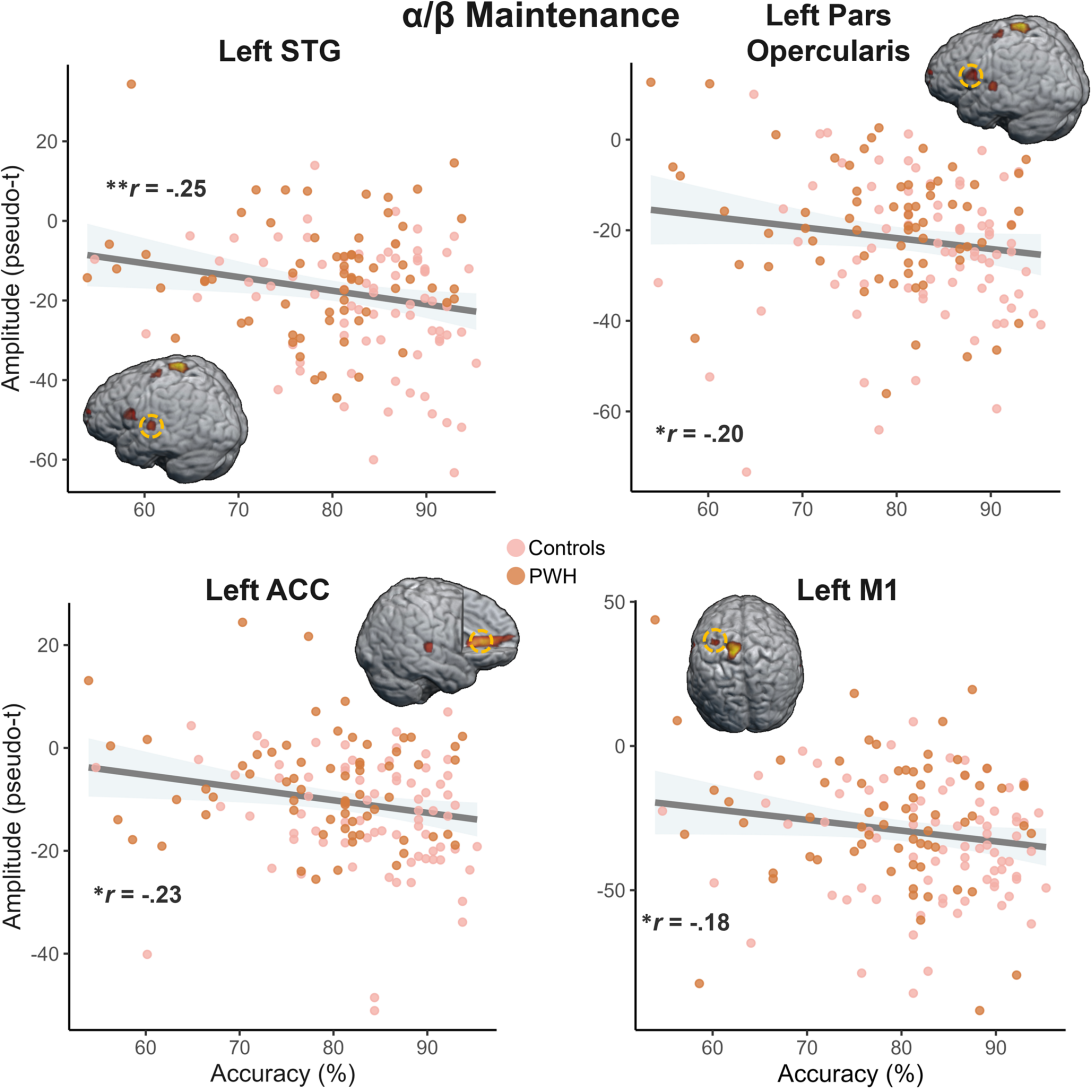
Neurobehavioral relationships. Stronger (i.e., more negative) alpha/beta oscillations during memory maintenance were significantly correlated with better task accuracy across both groups in the left superior temporal gyrus (STG), pars opercularis, anterior cingulate cortex (ACC), and primary motor cortex (M1) regardless of group. The gray shading around the trendlines represents the standard error of the mean. The accompanying r values were derived from partial correlation controlling for age. *p < .05, **p < .005

To evaluate neurobehavioral relationships, oscillatory responses were averaged across the four encoding windows for each peak identified in the mixed-model ANCOVA. Peaks of significant clusters were also extracted from the maintenance phase. These data points were then correlated with task performance via partial correlation controlling for age to examine the relationship between alpha/beta activity and VWM ability. Across both groups, activity during the maintenance period in the left STG (*r* = − .25, *p* = .003), pars opercularis (*r* = − .20, *p* = .019), ACC (*r* = − .23, *p* = .006), and M1(*r* = − .18, *p* = .036) were significantly correlated with task accuracy, such that stronger (i.e., more negative) alpha/beta oscillations were coupled to increased accuracy (Fig. 7). Four control participants and one PWH were excluded from the maintenance correlation analyses for having average peak activity values 3 SDs from the mean. Importantly, the identified peaks, except for the right STG and left M1, remained significant at *p* < .005 after removing these participants from the maintenance analyses.

**Fig. 8:**
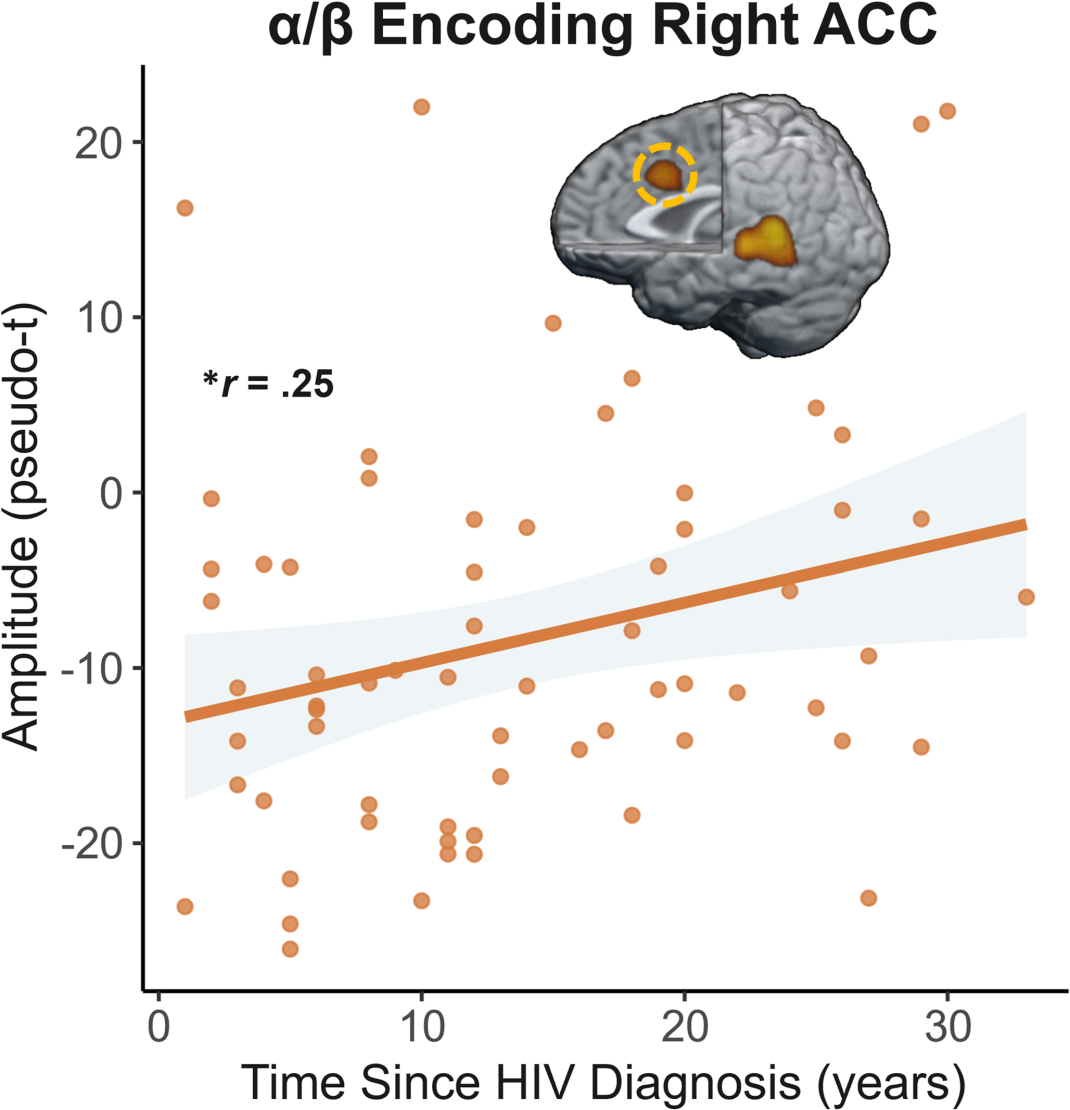
Disease duration is related to oscillatory activity during encoding. Weaker alpha/beta oscillations (i.e., less negative) in the right anterior cingulate cortex (ACC) throughout the encoding period was significantly correlated with longer time since HIV diagnosis among PWH above and beyond the effects of age. The gray shading around the trendline represents the standard error of the mean. The *r* value displayed was derived from partial correlation controlling for age. *p < .05

To evaluate the relationships with clinical indices of HIV, the extracted peaks were correlated with the time since HIV diagnosis, duration on ART, and current CD4 count controlling for age. Time since HIV diagnosis was positively correlated with average alpha/beta activity in the right ACC (*r* = .25, *p* = .045), such that longer time since diagnosis was associated with weaker (i.e., more positive) alpha/beta oscillations during encoding above and beyond the effects of age (Fig. 8).

## Discussion

Herein, we examined the impact of HIV status on task performance and the oscillatory dynamics serving VWM, over and above the effects of age. In accordance with previous MEG investigations of VWM, we observed robust alpha/beta range oscillations beginning in the visual cortices and extending to temporal and frontal regions throughout encoding and into maintenance across both groups, followed by a transition in posterior occipital cortices toward an increase in alpha/beta power compared to baseline during maintenance (Heinrichs-Graham and Wilson 2015; Killanin et al. 2022; Proskovec et al. 2016, 2019; Springer et al. 2023; Wilson et al. 2017). In line with our hypotheses, PWH exhibited weaker (i.e., less negative) alpha/beta oscillations during encoding in the left IFG and STG, two brain regions that are critical for executive and phonological VWM processing, respectively. These effects were driven by progressively larger group differences in the later time windows. This pattern was also observed in the right ACC, with weaker alpha/beta responses being significantly correlated with longer disease durations among PWH, above and beyond the effects of age. During the maintenance phase, PWH also exhibited weaker activity in the alpha/beta frequency range compared to control participants in the STG, ACC, cerebellum, M1, parietal, and left IFG, with stronger activity in the left STG, ACC, M1, and IFG being linked with better task performance. Finally, as predicted, PWH were significantly less accurate than controls on the VWM paradigm. Our findings indicate that the ability to fully encode and maintain verbal information for later retrieval is vulnerable to HIV status independent of age.

As discussed previously, the phonological loop is an integral part of Baddeley and Hitch’s model of working memory, tasked with storing language-based representations and playing a key role in the rehearsal component of WM processing (Baddeley and Hitch 1974). Activation of this loop has been widely reported in both MEG and fMRI investigations of VWM (Habeck et al. 2005, 2022; Heinrichs-Graham and Wilson 2015; Proskovec et al. 2019; Rottschy et al. 2012; Springer et al. 2023), and broadly linked to task performance. Thus, the weaker alpha/beta responses exhibited by PWH in brain areas linked to the phonological loop (e.g., left STG) likely suggests degraded processing in this component of the system, which notably extended across both encoding and maintenance phases of the task. Such an interpretation is supported by our finding linking weaker alpha/beta oscillations in the left STG during memory maintenance to poorer performance (i.e., lower accuracy) across the whole sample.

Beyond the phonological loop, PWH also exhibited reduced alpha/beta responses in the left IFG and ACC during both encoding and maintenance, which have been broadly linked to the so-called central executive component of WM processing (Kim 2019; Li et al. 2022; Wilson et al. 2016). The left IFG, particularly the pars opercularis, has been associated with executive function during language-based tasks and is very commonly reported region in VWM tasks (Courtney 2004; Deldar et al. 2020; Proskovec et al. 2016), with many studies suggesting that this region may extensively interact with the left STG in the midst of directing rehearsal operations to maintain representations in the phonological loop. Interestingly, our findings in the alpha/beta range also extended to the ACC, a critical region for attentional control that has also been widely implicated in WM processing (Li et al. 2022; MacDonald et al. 2000). Like the IFG, group differences in the ACC extended across both encoding and maintenance phases, and stronger neural oscillations in both regions during maintenance was coupled to better performance (i.e., higher accuracy), with the latter observation clearly supporting the view that the weaker responses in PWH were aberrant. Regarding the role of the ACC in the current task, we propose that it was likely attention or executive control based, as studies have shown that the ACC is equal or more active during object-based WM tasks compared to VWM paradigms (Assaf et al. 2006; Li et al. 2022).

A critically novel aspect of our investigation was the emphasis on the dynamic evolution of neural oscillatory responses. Generally, these data indicated that group differences became progressively larger during the encoding phase across key brain regions for VWM processing. Specifically, PWH exhibited responses similar to controls in both the left STG and ACC during the first 1000 ms of stimulus processing, but thereafter the strength of alpha/beta oscillations sharply increased in controls, while PWH exhibited much smaller increases (or no increases, e.g., in STG) over the remainder of the encoding period. This divergence in the second half of encoding also extended to the left IFG and may reflect that PWH were either unable to maintain strong activation levels, that a neuronal or cognitive resource ceiling was reached (e.g., limit on local neural populations synchronized in the alpha/beta range or limits in sustained attention), or another mechanism. Furthermore, alpha/beta encoding activity in the ACC was significantly correlated with disease duration when controlling for age among PWH in the current study, indicating that those with the longest disease durations had the weakest alpha/beta oscillations in this area (i.e., most abnormal). This may suggest greater difficulty in fully engaging these task-related neural populations in those with more extended disease durations. These findings could also suggest a breakdown in functional connectivity between regions, which could impact rehearsal operations and ultimately lead to degraded behavioral performance, as was observed among PWH. Future studies using dynamic neuroimaging approaches will be needed to further interrogate the neural time series to distinguish these alternatives.

Regarding how our findings map onto previous neuroHIV work, the key brain regions and time course are mostly consistent with those reported in Wilson et al. (2017), which used a very similar task in older PWH and controls. However, there are also some discrepancies, as our most robust differences were during the second half of encoding and early maintenance, while Wilson and colleagues found sustained group differences throughout maintenance and no differences during encoding. Further, their group differences during the maintenance phase included stronger left hemispheric alpha oscillations (i.e., hyper-activation) in PWH, which is reverse to that observed in the current study during late encoding and early maintenance. Interestingly, the key findings of the Wilson et al. (2017) study were during the latter maintenance phase, where we did not observe any significant responses in our sensor-level analyses. Moreover, in contrast to the current study, Wilson et al. did not examine the transient increase in alpha/beta power during early maintenance, choosing to focus instead on the lower frequency responses that emerged later in the time course. These disparate results could be attributable to many different factors. For one, the age range in Wilson et al. was 50–70 years old with a mean near 58 years in both groups, while the current study had a range of 20–66 years old and a mean near 46 years. However, we covaried out the effect of aging in the current study, which should have minimized any such effects. A second possible explanation for the directionality differences is that the mean neural responses in the left STG and IFG appear to be about twice as large in controls in the current study compared to Wilson et al., while the PWH have similar responses. This could reflect that the older controls relied more on right hemispheric homologue regions for VWM processing, which would be consistent with the authors’ interpretation in their 2017 paper and key tenets of the CRUNCH hypothesis. A third possibility is that these disparate findings simply reflect greater statistical power in the current study, as our group sizes were much larger than the 18 participants per group reported in Wilson et al. However, caution is warranted as there are many other possible factors that may have contributed. Regardless, the current findings are a strong compliment to those of Wilson et al., as the two studies have important differences and are mutually informative.

Before closing, it is necessary to address some of the limitations of this investigation. First, while our controls and PWH were matched on many demographic variables, we did not assess all possible variables, and it is likely that there were differences on some less common measures. This could have impacted VWM performance and neural activity, slightly biasing indices one way or another. Second, only VWM was addressed as opposed to other forms of WM that have been shown to be impacted by HIV, such as spatial and visual WM (Egan 1992; Martin et al. 2018). Future work should examine the differential oscillatory dynamics between PWH and seronegative controls during these other forms of WM to determine the extent of impairment. Finally, future work should consider a broader range of clinical measures, including inflammatory levels and other indices such as mitochondrial function that are known to be persistently affected in PWH, even during prolonged viral suppression. We feel the latter is especially important given recent findings that such inflammatory levels can affect both cognitive and brain function (Dietz et al. 2023; Spooner et al. 2023, 2024; Spooner, Taylor, Ahmad, Spooner et al. 2021a, b; Spooner, Taylor, Moshfegh, Spooner et al. 2021a, b).

In this investigation, we leveraged the spatiotemporal precision of MEG to dissociate the encoding and maintenance phases of VWM, and to derive the dynamics within the separate phases. This approach enabled us to quantify the persistent engagement of neural populations supporting WM encoding and maintenance operations and to determine that oscillations within these neural populations were much weaker in PWH. Further, we found that the strength of such oscillations in several regions was coupled to behavioral performance across all participants, and that HIV disease duration was correlated with such oscillatory responses in the right ACC. Future studies should examine a broader range of HIV clinical metrics in the context of VWM performance and brain dynamics, as well as consider the impact of factors known to be persistently abnormal in PWH (e.g., persistent inflammation, altered mitochondrial redox environments, etc.).

## Supplementary Information

Below is the link to the electronic supplementary material.

Supplementary Material 1

## Data Availability

The data used in this article will be made publicly available through the COINS framework at the completion of the study (https://coins.trendscenter.org/).
