# Supplementary material for: Oscillatory Dynamics Serving Verbal Working Memory Differ in People with HIV and Are Linked To Disease Duration
Source: J Neuroimmune Pharmacol. 2025 Aug 19;20(1):77. doi: 10.1007/s11481-025-10235-0 (PMC12364757; doi:10.1007/s11481-025-10235-0)
Supplement: Supplementary file 1 — Supplementary Material 1 [file 11481_2025_10235_MOESM1_ESM.docx]

**Supplemental Materials**


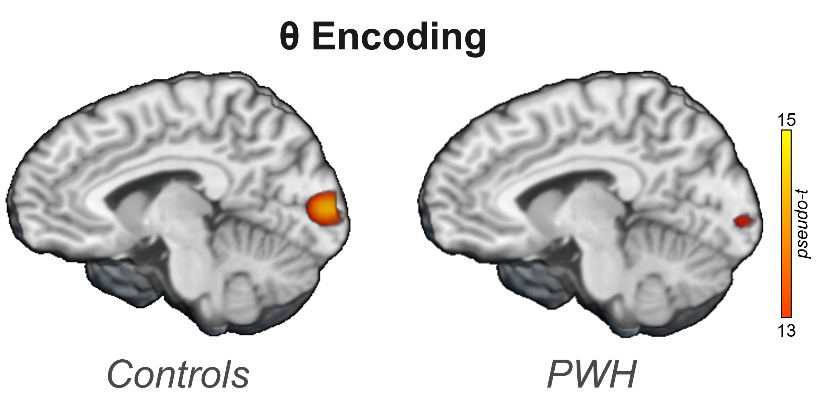
***Oscillatory Dynamics Serving Verbal Working Memory Differ in People with HIV and are Linked to Disease Duration (McDonald et al.)***

**Figure S1. Oscillatory theta activity during the encoding period by group.** Theta activity during encoding (0 to 400 ms) was observed in the visual cortices across both controls and PWH.


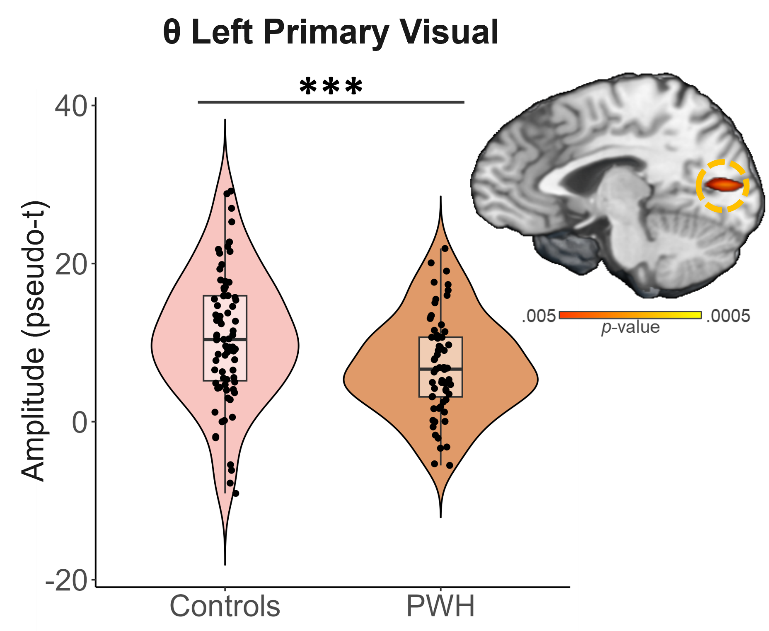


**Figure S2. HIV status modulates the visual theta response during working memory.** Whole-brain, one-way ANCOVA controlling for age revealed a significant group difference in theta activity within the left primary visual cortex. The statistical brain map is shown with a violin plot depicting group differences in amplitude. A color scale bar beneath the brain map shows the corresponding *p*-values. ****p* < .001
